# LMO1 polymorphisms and the risk of neuroblastoma: Assessment of meta‐analysis of case‐control studies

**DOI:** 10.1111/jcmm.14836

**Published:** 2019-12-12

**Authors:** Mohammad Hashemi, Sahel Sarabandi, Shima Karami, Jarosław Śmieja, Abdolkarim Moazeni‐Roodi, Saeid Ghavami, Marek J. Łos

**Affiliations:** ^1^ Genetics of Non‐communicable Disease Research Center Zahedan University of Medical Sciences Zahedan Iran; ^2^ Department of Clinical Biochemistry School of Medicine Zahedan University of Medical Sciences Zahedan Iran; ^3^ Institute of Automatic Control Silesian University of Technology Gliwice Poland; ^4^ Department of Clinical Biochemistry Iranshahr University of Medical Sciences Iranshahr Iran; ^5^ Department of Human Anatomy and Cell Science Rady Faculty of Health Sciences Max Rady College of Medicine University of Manitoba Winnipeg MB Canada; ^6^ CancerCare Manitoba Research Institute in Oncology and Hematology University of Manitoba Winnipeg MB Canada; ^7^ Biotechnology Centre Silesian University of Technology Gliwice Poland

**Keywords:** LMO1, meta‐analysis, neuroblastoma, polymorphism

## Abstract

Neuroblastoma (NB), a neuroendocrine tumour, is one of the most prevalent cancers in children. The link between LMO1 polymorphisms and NB has been investigated by several groups, rendering inconclusive results. Here, with this comprehensive systematic review and up‐to‐date meta‐analysis, we aim to distinctively elucidate the possible correlation between LMO1 polymorphisms and NB susceptibility. Eligible studies were systematically researched and identified using PubMed, Web of Science and Scopus databases up to 10 February 2019. Odds ratios (ORs) and 95% confidence intervals (CIs) were calculated to assess the strength of the associations. Our findings revealed that rs110419 and rs2168101 polymorphisms were significantly associated with a decreased risk of NB in all genetic models. In addition, the rs4758051 variant appeared protective against NB in homozygous, dominant and allele genetic models, whereas the rs10840002 variant markedly decreased the risk of NB in the allele model. In contrast, the rs204938 polymorphism showed a positive association with NB susceptibility in allele genetic models. In summary, our meta‐analysis is the first to provide clear evidence of an association between specific polymorphisms of LMO1 and susceptibility to NB. Of note, additional larger well‐designed studies would be helpful to further evaluate and confirm this association.

## 1 INTRODUCTION

Neuroblastoma (NB) is the most common solid tumour outside of the cranium in children, especially within the first 5 years after birth (median age of diagnosis at about 17 months).^1^, ^2^, ^3^ The tumours are most common in the abdomen (65%), followed by the neck, pelvis and chest (2). Neuroblastoma is a neuroendocrine tumour, which originates from the developing sympathetic nervous system, and its prevalence varies worldwide, affecting approximately 8‐14 individuals per million in the developed countries.^4^

Possible risk factors suspected of aiding the development of NB in children include parental exposure to radiation sources, solders, wood dust and hydrocarbons.^5^, ^6^ Hence, degradation of environment may contribute to the occurrence of the cancer. Furthermore, with the advances in regenerative medicine and the use of novel biomaterials in implants such risks may increase.^7^, ^8^

Our group performed in the past years several meta‐analyses,^9^, ^10^, ^11^ which underlined the role of polymorphisms in various cancer‐associated genes. Over the last decade, genome‐wide association studies (GWAS) have identified several loci linked to NB susceptibility,^12^, ^13^, ^14^, ^15^, ^16^, ^17^, ^18^, ^19^, ^20^, ^21^, ^22^ of which the LIM domain only 1 (LMO1) gene at 11p15.4 represents a promising candidate.^14^ LMO1 was recognized as neuroblastoma oncogene.^14^ It also acts as an oncogene in colorectal cancer (CRC) and lung cancer. LMO1 overexpression is a new predictive marker for anti‐EGFR therapy.^23^, ^24^ However, no significant differences were observed for LMO1 gene expression level between tumour tissues and corresponding adjacent benign tissues in human breast cancer, hepatocellular carcinoma (HCC) and gastric cancer (GC), which suggests that LMO1 gene may display a more complex functional network in these cancers.^24^ Sun et al^25^ have found that the expression levels of LMO1 in gastric cancer tissues were higher than those in adjacent tissues and the overexpression of LMO1 could be as a markers of poor prognosis. Deregulated expression of LMO1 may be involved in the development and maintenance of T‐ALL (T‐acute lymphoblastic leukaemia).^26^

Thus far, several studies have investigated LMO1 polymorphisms and their impact on NB susceptibility, with varying and inconclusive results.^14^, ^20^, ^27^, ^28^, ^29^, ^30^, ^31^, ^32^, ^33^ In the current study, we performed an up‐to‐date meta‐analysis to more precisely evaluate the association between specific LMO1 polymorphisms and NB susceptibility.

## 2 METHODS

### 2.1 Literature search

To identify all potentially eligible literature, PubMed, Scopus and Web of Science databases were searched for relevant publications up to February 2019. The search keywords were ‘neuroblastoma’ and ‘LIM domain only 1 or LMO1’ and ‘polymorphism or mutation or variation’. Studies were included in our meta‐analysis if they met the following inclusion criteria: (a) original case‐control studies; and (b) studies comprising necessary genotyping data of LMO1 polymorphisms in both disease cases and controls. The exclusion criteria were as follows: (a) case reports, conference abstracts, meta‐analyses and duplication data; and (b) studies lacking genotype information.

### 2.2 Data extraction

Two investigators independently searched literature and extracted the appropriate data from eligible studies. Data collected from each study included: the first author, publication date, country, ethnicity of study participants, control‐population source, genotyping methods of LOM1 polymorphisms, genotype distributions in cases and controls, and the result of the HWE test (Table 1).

**Table 1:** Characteristics of all studies included in the meta‐analysis

First author	Year	Country	Ethnicity	Source of control	Genotyping method	Case/Control	Cases					Controls					HWE (P)
rs110419							AA	AG	GG	A	G	AA	AG	GG	A	G	
Capasso M	2013	Italy	Caucasian	PB	Illumina HumanHap550	323/774	87	152	84	326	320	133	370	271	636	912	0.727
Capasso M	2013	USA	European American	PB	Illumina HumanHap550	1626/2575	509	787	330	1805	1447	599	1310	666	2508	2642	0.357
He J	2016	China	Asian	HB	TaqMan	256/531	103	117	36	323	189	159	275	97	593	469	0.248
He L	2018	China	Asian	HB	TaqMan	313/762	150	118	45	418	208	279	355	128	913	611	0.405
Latorre V	2012	USA	African American	HB	Illumina HumanHap 550	365/2491	223	124	18	570	160	1491	863	137	3845	1137	0.409
Lu J	2015	China	Asian	HB	MassARRAY iPLEX	244/305	–	–	–	359	129	–	–	–	369	241	–
Oldridge DA	2015	USA	European American	N.A	Illumina HumanHap550	2101/4202	–	–	–	2349	1853	–	–	–	4110	4294	–
Wang K	2011	USA	Discovery	N.A	Illumina HumanHap550	1627/3254	–	–	–	1790	1464	–	–	–	3189	3319	–
Wang K	2011	USA	US replication	N.A	Illumina Human610	190/1507	–	–	–	232	148	–	–	–	1477	1537	–
Wang K	2011	USA	UK replication	N.A	TaqMan	253/845	–	–	–	268	238	–	–	–	811	879	–
Wang K	2011	USA	Italian replication	N.A	TaqMan	181/491	–	–	–	177	185	–	–	–	403	579	–
Zhang J	2017	China	Asian	HB	TaqMan	374/812	150	171	53	471	277	245	417	150	907	717	0.239
rs4758051							GG	AG	AA	G	A	GG	AG	AA	G	A	
Capasso M	2013	Italy	Caucasian	PB	Illumina HumanHap550	340/792	70	156	114	296	384	141	405	246	687	897	0.248
Capasso M	2013	USA	European American	PB	Illumina HumanHap550	1624/2571	436	787	401	1659	1589	525	1292	754	2342	2800	0.507
He J	2016	China	Asian	HB	TaqMan	256/531	95	126	35	316	196	194	242	95	630	432	0.199
He L	2018	China	Asian	HB	TaqMan	313/762	138	123	52	399	227	256	364	142	876	648	0.530
Latorre V	2012	USA	African American	HB	Illumina HumanHap 550	365/2491	239	108	18	586	144	1692	713	86	4097	885	0.310
Lu J	2015	China	Asian	HB	MassARRAY iPLEX	244/305	–	–	–	332	156	–	–	–	357	253	–
Oldridge DA	2015	USA	European American	N.A	Illumina HumanHap550	2101/4202	–	–	–	2059	2143	–	–	–	4605	3799	–
Wang K	2011	USA	Discovery	N.A	Illumina HumanHap550	1627/3254	–	–	–	1660	1594	–	–	–	2929	3579	–
Wang K	2011	USA	US replication	N.A	Illumina Human610	190/1507	–	–	–	209	171	–	–	–	1356	1658	–
Wang K	2011	USA	UK replication	N.A	TaqMan	253/845	–	–	–	258	248	–	–	–	761	930	–
Wang K	2011	USA	Italian replication	N.A	TaqMan	181/491	–	–	–	163	199	–	–	–	412	570	–
Zhang J	2017	China	Asian	HB	TaqMan	374/812	145	185	44	475	273	282	380	150	944	680	0.271
rs10840002							AA	AG	GG	A	G	AA	AG	GG	A	G	
He J	2016	China	Asian	HB	TaqMan	256/531	90	124	42	304	208	182	240	109	604	458	0.070
He L	2018	China	Asian	HB	TaqMan	313/762	120	128	65	368	258	240	375	147	855	669	0.981
Latorre V	2012	USA	African American	HB	Illumina HumanHap 550	365/2491	204	128	33	536	194	1430	897	164	3757	1225	0.148
Lu J	2015	China	Asian	HB	MassARRAY iPLEX	244/305	–	–	–	317	171	–	–	–	342	268	–
Wang K	2011	USA	Discovery	N.A	Illumina HumanHap550	1627/3254	–	–	–	1367	1887	–	–	–	2408	4100	–
Wang K	2011	USA	US replication	N.A	Illumina Human610	190/1507	–	–	–	167	213	–	–	–	1145	1869	–
Wang K	2011	USA	UK replication	N.A	TaqMan	253/845	–	–	–	187	319	–	–	–	608	1082	–
Zhang J	2017	China	Asian	HB	TaqMan	374/812	132	186	56	450	298	260	384	168	904	720	0.233
rs204938							AA	AG	GG	A	G	AA	AG	GG	A	G	
He J	2016	China	Asian	HB	TaqMan	256/531	164	83	9	411	101	354	165	12	873	189	0.153
He L	2018	China	Asian	HB	TaqMan	313/762	200	97	16	497	129	476	258	28	1210	314	0.336
Latorre V	2012	USA	African American	HB	Illumina HumanHap 550	365/2490	42	162	161	246	484	241	1040	1209	1522	3458	0.426
Lu J	2015	China	Asian	HB	MassARRAY iPLEX	244/305	–	–	–	359	129	–	–	–	489	121	–
Wang K	2011	USA	Discovery	N.A	Illumina HumanHap550	1627/3254	–	–	–	1660	1594	–	–	–	3644	2864	–
Wang K	2011	USA	US replication	N.A	Illumina Human610	190/1507	–	–	–	190	190	–	–	–	1658	1356	–
Wang K	2011	USA	UK replication	N.A	TaqMan	253/845	–	–	–	253	253	–	–	–	946	744	–
Zhang J	2017	China	Asian	HB	TaqMan	374/812	241	119	14	601	147	522	262	28	1306	318	0.485
rs2168101							GG	GT	TT	G	T	GG	GT	TT	G	T	
He J	2018	China	Asian	HB	TaqMan	373/812	245	117	11	607	139	407	342	63	1156	468	0.448
He L	2018	China	Asian	HB	TaqMan	313/762	214	85	14	513	113	401	310	51	1112	412	0.389
Oldridge DA	2015	USA	European American	N.A	Illumina HumanHap550	–	–	–	–	3185	1017	–	–	–	5774	2630	–

### 2.3 Statistical analysis

All analyses were performed using STATA 14.1 (Stata Corporation). Departure from Hardy‐Weinberg equilibrium (HWE) in controls was examined by the *χ* ^2^ test. The strength of the association between LMO1 polymorphisms and NB risk was assessed by pooled odds ratios (ORs) and their 95% confidence intervals (CIs). The *Z*‐test was implemented to establish the statistical significance of the pooled ORs. We estimated the between‐study heterogeneity by the *Q*‐test and *I* ^2^‐test, with *P* < .10 indicating the presence of heterogeneity. In case of heterogeneity, a random‐effect model was used; otherwise, a fixed‐effect model was employed.

We determined publication bias using funnel plots for visual inspection and by conducting quantitative estimations using the Egger's test. Sensitivity analyses were carried out by sequentially ignoring a single study at a time to assess the impact of individual data sets on the pooled ORs.

## 3 RESULTS

### 3.1 Study characteristics

**Figure 1:**
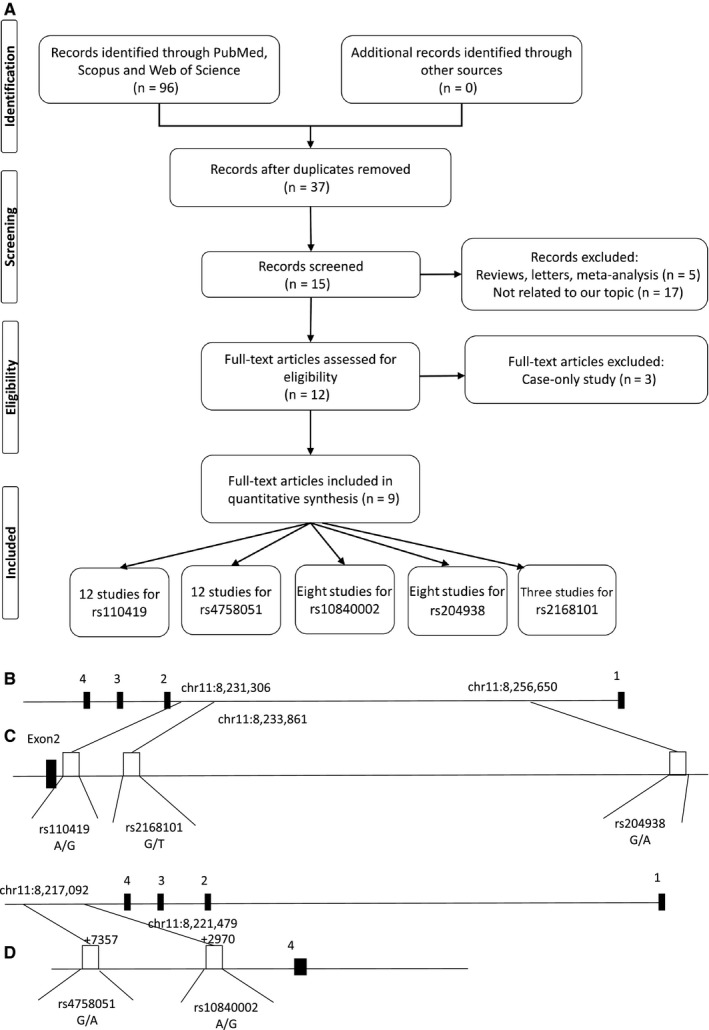
Basic information about the presented study. (A) Flow chart of the study selection procedure, (B) map of the human LMO1 gene (USCS genome browser: chr11:8,224,449‐8,263,388). Exons 1‐4 are numbered and represented by black boxes. C) Positions of the single‐nucleotid variations within the first intron of the LMO1 gene (D) positions of the single‐nucleotid variations within the 3′ UTR region of the LMO1 gene (not up to scale)

Figure 1A shows a flow chart of the study selection procedure. Ultimately, 9 published articles^14^, ^20^, ^27^, ^28^, ^29^, ^30^, ^31^, ^32^, ^33^ that met our inclusion criteria were identified: 12 case‐control studies on rs110419 and rs4758051 polymorphisms, 8 studies on rs10840002 and rs204938 polymorphisms, and three studies on the rs2168101polymorphism were also included in our meta‐analysis. The Figure 1B‐D illustrates the position of the analysed polymorphisms within the LMO1 gene. The articles were published between 2011 and 2018, and they include representatives of major ethnic groups (Caucasians, European Americans, African Americans and Asians). The main characteristics of these studies are listed in Table 1.

#### 3.1.1 Association of rs110419 polymorphism and neuroblastoma risk

**Figure 2:**
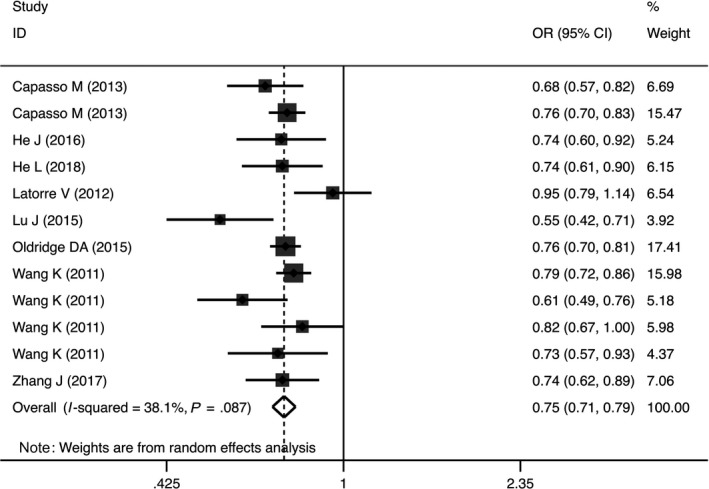
Forest plot representing the association between the LMO1 rs110419 polymorphism and neuroblastoma susceptibility in allele genetic models (G vs A)

Quantitative analysis revealed that the rs110419 variant markedly decreased the risk of NB in heterozygous (OR = 0.72, 95%CI = 0.65‐0.79, *P* < .00001, AG vs AA), homozygous (OR = 0.59, 95%CI = 0.52‐0.67, *P* < .00001, GG vs AA), dominant, (OR = 0.68, 95%CI = 0.59‐0.78, *P* < .00001, AG + GG vs AA), recessive (OR = 0.73, 95%CI = 0.66‐0.82, *P* < .00001, GG vs AG + AA) and allele (OR = 0.75, 95%CI = 0.71‐0.79, *P* < .00001, G vs A) genetic models (Table 2, Figure 2).

**Table 2:** Association between LMO1 polymorphisms and susceptibility to neuroblastoma

Polymorphism	No.	Genetic model	Test of association			Heterogeneity (*I* ^2^ (%), *P*)			Egger's test
			OR (95%CI)	*Z*	*P*	*χ* ^2^	*I* ^2^ (%)	*P*	*P*
rs110419	6	AG vs AA	0.72 (0.65‐0.79)	6.77	<.00001	8.15	39	.15	.643
	6	GG vs AA	0.59 (0.52‐0.67)	8.09	<.00001	4.03	0	.55	.565
	6	AG + GG vs AA	0.68 (0.59‐0.78)	5.29	<.00001	10.65	53	.06	.772
	6	GG vs AG + AA	0.73 (0.66‐0.82)	5.51	<.00001	1.66	0	.89	.411
	12	G vs A	0.75 (0.71‐0.79)	10.14	<.00001	17.78	38	.09	.293
rs4758051	6	AG vs GG	0.85 (0.71‐1.01)	1.85	.06	13.56	63	.02	.487
	6	AA vs GG	0.76 (0.61‐0.96)	2.32	.02	12.36	60	.03	.207
	6	AG + AA vs GG	0.83 (0.70‐0.99)	2.08	.04	15.62	68	.008	.363
	6	AA vs AG + GG	0.86 (0.70‐1.06)	1.38	.17	13.51	63	.02	.612
	12	A vs G	0.86 (0.75‐0.99)	2.13	.03	121.1	91	<.00001	.245
rs10840002	4	AG vs AA	0.92 (0.80‐1.05)	1.24	.22	9.98	40	.17	.764
	4	GG vs AA	0.89 (0.65‐1.23)	0.71	.48	8.04	63	.05	.750
	4	AG + GG vs AA	0.91 (0.80‐1.04)	1.35	.18	4.38	32	.22	.506
	4	GG vs AG + AA	0.94 (0.68‐1.30)	0.37	.71	9.98	70	.02	.724
	8	G vs A	0.87 (0.79‐0.95)	3.00	.003	15.31	54	.03	.587
rs204938	4	AG vs AA	0.96 (0.83‐1.12)	0.48	.63	0.97	0	.81	.922
	4	GG vs AA	0.97 (0.74‐1.28)	0.21	.83	4.11	27	.25	.044
	4	AG + GG vs AA	0.97 (0.84‐1.13)	0.36	.72	1.76	0	.62	.685
	4	GG vs AG + AA	0.92 (0.76‐1.12)	0.79	.43	4.20	28	.24	.046
	8	G vs A	1.13 (1.00‐1.26)	2.03	.04	20.15	65	.005	.635
rs2168101	2	GT vs GG	0.54 (0.45‐0.66)	6.13	<.00001	0.25	0	.61	–
	2	TT vs GG	0.39 (0.25‐0.60)	4.17	<.00001	1.56	36	.21	–
	2	GT + TT vs GG	0.52 (0.43‐0.63)	6.84	<.00001	0.01	0	.91	–
	2	TT vs GT + GG	0.48 (0.31‐0.75)	3.21	.001	1.70	41	.19	
	3	G vs T	0.64 (0.55‐0.74)	5.96	<.00001	4.54	56	.10	–

**Figure 3:**
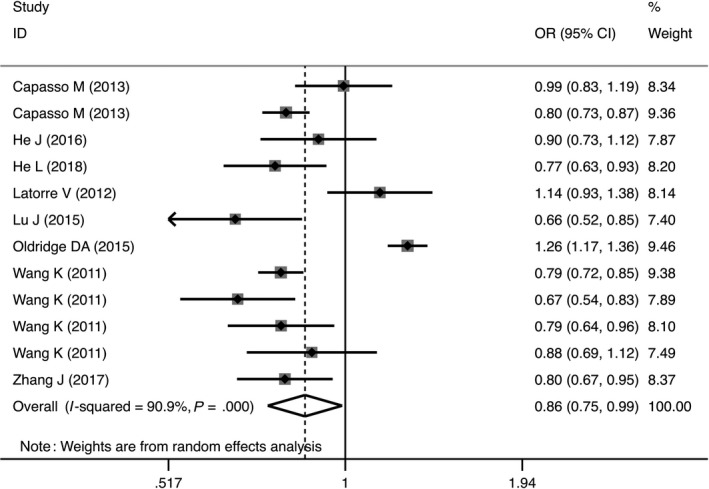
Forest plot representing the association between the LMO1 rs4758051 polymorphism and neuroblastoma susceptibility in allele genetic models (A vs G)

The rs4758051 variant markedly decreased the risk of NB in homozygous (OR = 0.76, 95%CI = 0.61‐0.96, *P* = .02, AA vs GG), dominant (OR = 0.68, 95%CI = 0.59‐0.78, *P* = .04, AG + GG vs AA) and allele (OR = 0.86, 95%CI = 0.75‐0.99, *P* = .03, A vs G) genetic models (Table 2, Figure 3). Similar findings were true for the rs10840002 variant, but only in the allele genetic model OR = 0.87, 95%CI = 0.79‐0.95, *P* = .003, G vs A; Table 2). In addition, the rs2168101 polymorphism was associated with decreased risk of NB susceptibility in heterozygous (OR = 0.54, 95%CI = 0.45‐0.66, *P* < .00001, GT vs GG), homozygous (OR = 0.39, 95%CI = 0.25‐0.60, *P* < .00001, TT vs GG), dominant (OR = 0.52, 95%CI = 0.43‐0.63, *P* < .00001, GT + TT vs GG), recessive (OR = 0.48, 95%CI = 0.31‐0.75, *P* = .001, TT vs GT + GG) and allele (OR = 0.64, 95%CI = 0.55‐0.74, *P* < .00001, G vs T) genetic models (Table 2). In contrast to the other polymorphisms evaluated, the results revealed that rs204938 marginally increased the risk of NB in the allele genetic model (OR = 1.13, 95%CI = 1.00‐1.26, *P* = .04, G vs A; Table 2).

#### 3.1.2 Heterogeneity and publication bias

Between‐study heterogeneity across studies included into pooled analysis is displayed in Table 2. No evidence of heterogeneity was observed between studies for rs110419 and rs2168101 polymorphisms. For rs4758051; however, heterogeneity was identified in all codominant, dominant, recessive and allele genetic models (Table 2). Regarding rs10840002, no heterogeneity was observed in heterozygous, homozygous and dominant genetic models. No evidence of heterogeneity was found for rs204938 in heterozygous, homozygous, dominant and recessive genetic models (Table 2).

**Figure 4:**
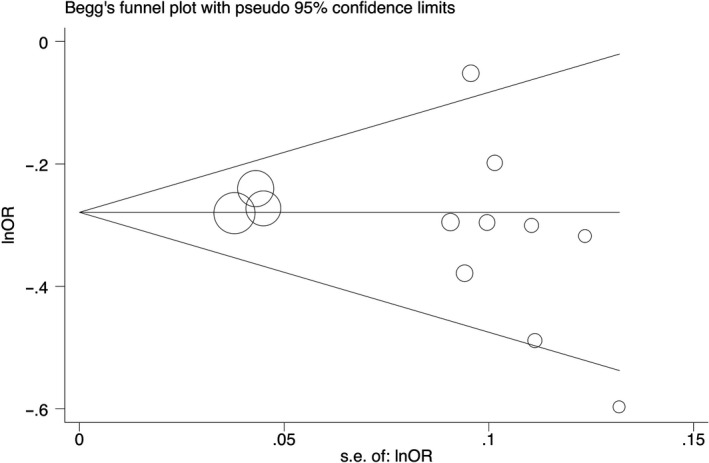
Begg's funnel plot for the association between the LMO1 rs110419 polymorphism and neuroblastoma risk (G vs A)

Begg's funnel plots and Egger's tests were performed to estimate the publication bias of the included literature. The Egger's tests revealed no existence of publication bias for all polymorphisms, except rs204938 in homozygous and recessive genetic models (Table 2, Figure 4).

#### 3.1.3 Sensitivity analysis

**Figure 5:**
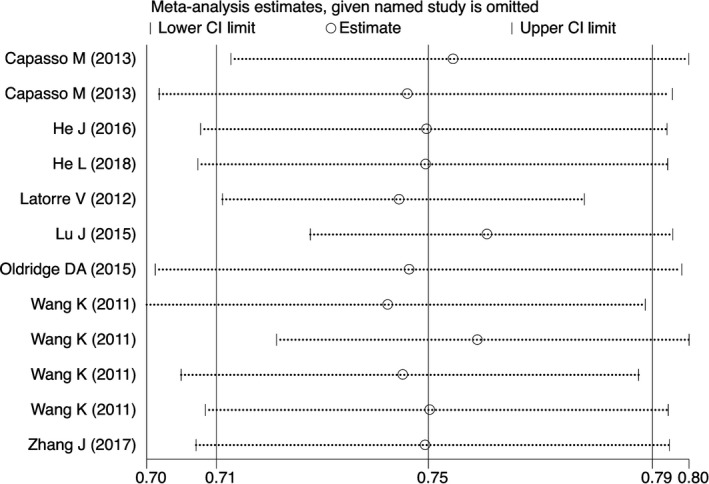
Sensitivity analyses of studies on the association of the LMO1 rs110419 polymorphism and neuroblastoma (G vs A)

Sensitivity analysis was conducted to assess the effects of individual studies on the stability of the pooled ORs. With sequential removal of individual study results from the analysis for rs110419, the pooled ORs remained significantly consistent in heterozygous, homozygous, recessive, dominant and allele genetic models (Figure 5). With regards to rs10840002, the ORs remained unchanged in heterozygous and allele genetic models. Lastly, the pooled ORs changed in all genetic models for rs204938 and rs4758051 polymorphisms.

## 4 DISCUSSION

Genetic susceptibility to NB has led to growing attention of the studies focused on genetic variations. To date, several reports on the potential association between LMO1 polymorphisms and NB development have been published, but the findings were inconsistent. Somehow surprisingly, none of the polymorphisms are in the coding region of the LMO1 gene. Therefore, they do not result in any amino acid change. They seem not to be related to splicing variants either and; therefore, the nature of their association with susceptibility to NB remains elusive. Three polymorphisms: rs110419, rs2168101 and rs204938 are located in the intron 1, while rs4758051 and rs1084000 are in the intergenic region, beyond the last, fourth exon of the LMO1 gene. Hence, the analysed polymorphisms most likely affect regulatory mechanisms within the LMO1 gene.

Our meta‐analysis, based on systematically collected studies, aimed to obtain an accurate summary of the estimates of the strength of association between specific LMO1 gene polymorphisms and NB susceptibility, and, to our best knowledge, is the first to do so. We found that rs110419, rs4758051, rs10840002 and rs2168101 polymorphisms were associated with reduced susceptibility to NB, while the rs204938 polymorphism increased the risk of the disease.

He et al^30^ reported that rs110419, rs10840002, rs4758051 and rs2168101 polymorphisms of the LMO1 gene were associated with a decreased risk of NB in an eastern Chinese subpopulation. In addition, the rs2168101 and rs3750952 polymorphisms were markedly associated with decreased NB susceptibility in children from North and South China.^28^ Similarly, the LMO1 rs110419 A > G polymorphism was linked to a reduced NB risk in Southern Chinese children.^29^ A significant association between the rs204926 variant and NB susceptibility has been reported,^32^ and rs4758051 and rs10840002 polymorphisms were associated with decreased NB.^33^ Furthermore, a significant association between the rs110419 polymorphism and risk of NB was observed in an Italian population as well as European American children.^27^ Conversely, no significant associations between LMO1 polymorphisms and NB risk were observed in African Americans.^31^ While some studies indicate that frequently occurring polymorphisms at the LMO1 locus are strongly connected to susceptibility to developing NB.^14^ The observed differences in susceptibility, between populations, are likely due to the overall genetic background that modifies the LMO1 prone risk factors.

This meta‐analysis has a few limitations that should be considered. First, we have only included studies published in the English language. Second, there was significant heterogeneity among studies. There was also variation in study sample size, populations and ethnicity of participants, (please see Table 1 for details). Third, our findings were obtained with a relatively limited sample size and consequently, our conclusions are preliminary in nature. Fourth, the assessments of gene‐gene and gene/environment interactions were not performed despite some data suggest so.

In conclusion, our meta‐analysis is the first to provide evidence of an association between specific genetic polymorphisms of the LMO1 gene and susceptibility to NB. Further validation by well‐designed studies performed by international multicenter programme (addressing diverse ethnic populations) is needed to conclusively confirm the impact of specific LMOI polymorphisms on NB susceptibility and development. Unfortunately, at present we lack sufficient number of studies (studied populations) to reliably perform such analyses. Nevertheless, the presented analysis offers interesting insight into the analysed polymorphisms, as outlined above.

## CONFLICT OF INTEREST

The authors declare no conflict of interest. The funders had no role in the design of the study; in the collection, analyses, or interpretation of data; in the writing of the manuscript or in the decision to publish the results.

## AUTHOR CONTRIBUTIONS

M. Hashemi, S. Sarabandi, S. Karami, A. Moazeni‐Roodi, J. Śmieja: involved in conceptualization, data collection, validation, statistical analysis and manuscript writing (first draft); S. Ghavami and MJ Łos: formally analysed and finalized the manuscript.
